# Establishment of Transgenic Lines for Jumpstarter Method Using a Composite Transposon Vector in the Ladybird Beetle, *Harmonia axyridis*

**DOI:** 10.1371/journal.pone.0100804

**Published:** 2014-06-24

**Authors:** Hisashi Kuwayama, Hiroki Gotoh, Yusuke Konishi, Hideto Nishikawa, Toshinobu Yaginuma, Teruyuki Niimi

**Affiliations:** 1 Graduate School of Bioagricultural Sciences, Nagoya University, Chikusa, Nagoya, Japan; 2 Department of Entomology, Washington State University, Pullman, Washington, United States of America; Virginia Tech, United States of America

## Abstract

In this post-genomic era, genome-wide functional analysis is indispensable. The recent development of RNA interference techniques has enabled researchers to easily analyze gene function even in non-model organisms. On the other hand, little progress has been made in the identification and functional analyses of cis-regulatory elements in non-model organisms. In order to develop experimental platform for identification and analyses of cis-regulatory elements in a non-model organism, in this case, the ladybird beetle, *Harmonia axyridis*, we established transgenic transposon-tagged lines using a novel composite vector. This vector enables the generation of two types of insertion products (jumpstarter and mutator). The jumpstarter portion carries a transposase gene, while the mutator segment carries a reporter gene for detecting enhancers. The full-composite element is flanked by functional termini (required for movement); however, the mutator region has an extra terminus making it possible for the mutator to remobilize on its own, thus leaving an immobile jumpstarter element behind. Each insertion type is stable on its own, but once crossed, jumpstarters can remobilize mutators. After crossing a jumpstarter and mutator line, all tested G_2_ females gave rise to at least one new insertion line in the next generation. This jumping rate is equivalent to the P-element-mediated jumpstarter method in *Drosophila*. These established transgenic lines will offer us the ideal experimental materials for the effective screening and identification of enhancers in this species. In addition, this jumpstarter method has the potential to be as effective in other non-model insect species and thus applicable to any organism.

## Introduction

For the last two decades, our knowledge of gene function in non-model organisms has dramatically increased [1]. Methodological advances in RNA interference (RNAi) enabled researchers to address various biologically interesting phenomena via gene knock down even in non-model organisms [2], [3]. On the other hand, there are very few studies on gene function via over expression or ectopic expression in non-model organisms (but see [4]). Transgenic techniques have often been used for over/ectopic expression analyses [3], [5], [6]. However, limited knowledge of cis-regulatory elements including enhancers in non-model organisms prevents studies from further progress [3], [6], [7]. Enhancer-trapping is an excellent method to detect enhancers in the genome following the expression pattern of a reporter gene [8]. Although the generation of enhancer trap lines by microinjection is time-consuming and labor-intensive, the jumpstarter method allows transposon-tagged lines to be generated by simply crossing two lines, thereby enabling genome-wide assays. In *Drosophila*, thousands of enhancer-trap lines have been established based on this method [9], [10]. Thus, an essential technique for the genome-wide analysis of gene function is the jumpstarter method, which was initially developed for insertional mutagenesis in *Drosophila* [11], [12]. This approach allows transposon-tagged lines to be generated en mass by simply crossing a jumpstarter line, containing a *transposase* gene, with a mutator line, containing a transposable element consisting of two inverted terminal repeat (ITR) sequences (5′ and 3′). The transposon requires both elements–the transposase and two ITRs–for mobilization. However, this valuable method has so far been confined to a few model organisms, for example, in ascidians [13], insects [11], [12], [14], [15], [16], vertebrates [17], [18], [19] and plants [20], because of difficulties associated with the generation of a jumpstarter strain. In particular, an immobilized jumpstarter element has generally thought impossible to generate with a single transposon, with the exception of a special case in which an exogenous DNA fragment without a transposable element was integrated into a mouse genome. Furthermore, even in *Drosophila*, immobilized jumpstarter elements have been produced accidentally through imprecise excision by the P element itself [11], [12]. To overcome these problems, we applied the transgene stabilization method [21] to a composite *piggyBac* vector that simultaneously generates two elements that can be used for the jumpstarter technique. This transposon-based transformation vector has an additional 5′ ITR [21]. When 5′ and 3′ ITRs at both ends of the vector are used for transposition, the entire vector is integrated into the genome. By contrast, when the ITR in the middle of the vector is used, only one-half of the vector is integrated; in this case, generating a mutator designed for enhancer trapping. After the integration of the entire vector, the transposase acts between the 5′ and 3′ ITRs, and one-half of the vector is excised leaving the *transposase* gene with only one 5′ ITR, thereby immobilizing it.

In order to develop an experimental platform for future identification of various enhancers, here we established transgenic transposon-tagged lines in ladybird beetle, *Harmonia axyridis*. By crossing two established lines (jumpstarter line and mutator line) we will be able to generate various enhancer- trap lines in an efficient manner.

## Materials and Methods

### Insects

Laboratory stocks of *Harmonia axyridis* were derived from field collections in Aichi, Japan. They were reared as described by [22].

### Construction of piggyBac Vector

A *Bgl* II-*Asc* I double digested fragment containing *EGFP* with the nuclear localization signal (NLS) under the *Drosophila heat shock protein 27* (*Dmhsp27*) minimal promoter was isolated from pSL1180fa[tetO-hsp27mp-NLS-EGFP] as described in [23] and was filled in with a Klenow fragment, cloned into the filled-in site of *Asc* I of pBac[3xP3-DsRed] [24] and named pBac[3xP3-DsRed, hsp27mp-NLS-EGFP]. A *Sac* I-*Xba* I double digested fragment containing 3xP3-*ECFP* was isolated from pBac[3xP3-ECFPafm] [25] and was filled in with a Klenow fragment, cloned into the filled-in site of *Eco*R I of pBac[3xP3-DsRed, hsp27mp-NLS-EGFP] and named pBac[3xP3-DsRed, hsp27mp-NLS-EGFP, 3xP3-ECFP]. An *Ase* I fragment containing the *piggyBac transposase* under *Drosophila heat shock protein 70* (*Dmhsp70*) promoter from phsp-pBac [26] was filled in with a Klenow fragment, and cloned into the filled-in site of *Asc* I of pBac[3xP3-DsRed, hsp27mp-NLS-EGFP, 3xP3-ECFP]. This composite vector was named pBac(hsp70-transposase)::(3xP3-ECFP)::hsp27-EGFP::(3xP3-DsRed).

### Generation of Transgenic Ladybird Beetles

Transgenic ladybird beetles were generated by microinjection of 500 ng/µl composite vector without a helper plasmid into the posterior pole of embryos during the syncytium stage of development as described by [27]. The G_0_ adults were crossed with non-injected wild type adults. The G_1_ newly hatched larvae were examined under a fluorescent stereomicroscope. ECFP, DsRed and EGFP fluorescence was observed using a fluorescent stereomicroscope (MZ FLIII, Leica) equipped with a CFP, a DsRED and a GFP2 filter (Leica), respectively. Detected fluorescent red colors were converted to magenta using DPcontroller software (Olympus Optical Co.) in order to increase visibility for potential color blind readers.

### Inverse PCR

Inverse PCR for a mutator strain was performed as described by [27]. Genomic DNA were digested with *HaeIII* or *MspI*. PCR was performed using the primers described in [28].

### Cloning

Total RNA was extracted from 0 to 2-day-old *Harmonia* eggs with Trizol (Gibco BRL) according to the manufacture’s instructions. The first-strand cDNA was synthesized with SMART PCR cDNA Amplification Kit (Clontech) using 1 µg of total RNA. *Harmonia axyridis ribosomal protein 49* (*Ha-rp49*) cDNA fragments were amplified using rp49-1 and rp49-2 primer sets. The following degenerate primers were designed corresponding to highly conserved amino acid sequences found in Rp49 among *D*. *melanogaster* Rp49 (**U92431**), mouse Rp49 (**M23453**), rat Rp49 (**X06483**) and yeast Rp49 (**Y13134**).

Degenerate primers for *rp49.*

rp49-1: 5′-ACIAARMAITTYATIMGICA-3′
rp49-2: 5′-TGIGCIATYTCISCRCARTA-3′

(R, A and G; Y, T and C; S, C and G; M, C and A; I, inosine).

PCRs were performed using 2.5 µl of the 10-fold diluted first-strand cDNA, a pair of primers from the list above, and AmpliTaq Gold (Perkin Elmer).

### Sequencing and Sequence Analysis

The PCR product was subcloned into the *Eco*R V site of the pBluescript KS+ vector (Stratagene). The nucleotide sequences of the PCR products and the flanking regions around the restriction enzyme-digested fragments, which were inserted into the vectors during cloning procedures, were confirmed using the dideoxy chain-termination method by an automatic DNA sequencer (CEQ 2000XL; Beckman Coulter or DNA sequencer 3130 genetic analyser; Applied Biosystems). Sequence analysis was carried out using a DNASIS system (Hitachi Software Engineering).

### Sequence Accession Number

DDBJ/EMBL/GenBank accession numbers for *Ha-rp49* cDNA is **AB552923**.

### RT-PCR

*H*. *axyridis* 0-day-old pupa of a jumpstarter strain or a wild type was used for total RNA extractions. Total RNA was treated with 10 U DNase I for 30 min at 37°C. 500 ng of DNase I treated total RNA was used for first-strand cDNA syntheses as described above. A reaction without reverse transcriptase (RT) was performed with cDNA synthesis and was used as a negative control for the RT-PCR experiment. The PCR cycle numbers are 35 cycles for *transposase* and *Ha-rp49*. The following primers were used. *Ha-rp49* was used as an internal control. Effect of heat shock on transposase expression has also investigated in differential heat shock temperatures *H*. *axyridis* 0-day-old pupae of a jumpstarter strain were exposed to heat shock for 1 h at 25°C, 30°C, 35°C, 40°C and 45°C. Exposed pupae were used for RNA extraction.

Gene-specific primer for *transposase.*

RT-transposase: 5′-CATCGTTTTCTCGAAGTGTGGGCCG-3′
PLF: 5′-CTTGACCTTGCCACAGAGGACTATTAGAGG-3′

Gene-specific primer for *Ha-rp49.*

Ha-rp49-#1: 5′-GCGATCGCTATGGAAAACTC-3′
Ha-rp49-#2: 5′-TACGATTTTGCATCAACAGT-3′

### PCR for Analyzing Mobilization

PCR were performed using extracted genome DNA as template. Sequence of PLR was according to [28]. Primers HaG1 and HaG3 were designed from original genome integrated site of mutator.

HaG1: 5′-AAGCCGAAGTAACAATTGAAATGTCACTGC-3′
HaG3: 5′-TGATCGTGATGAAGCTTCTCCCTATGCTGC-3′

## Results and Discussion

**Figure 1:**
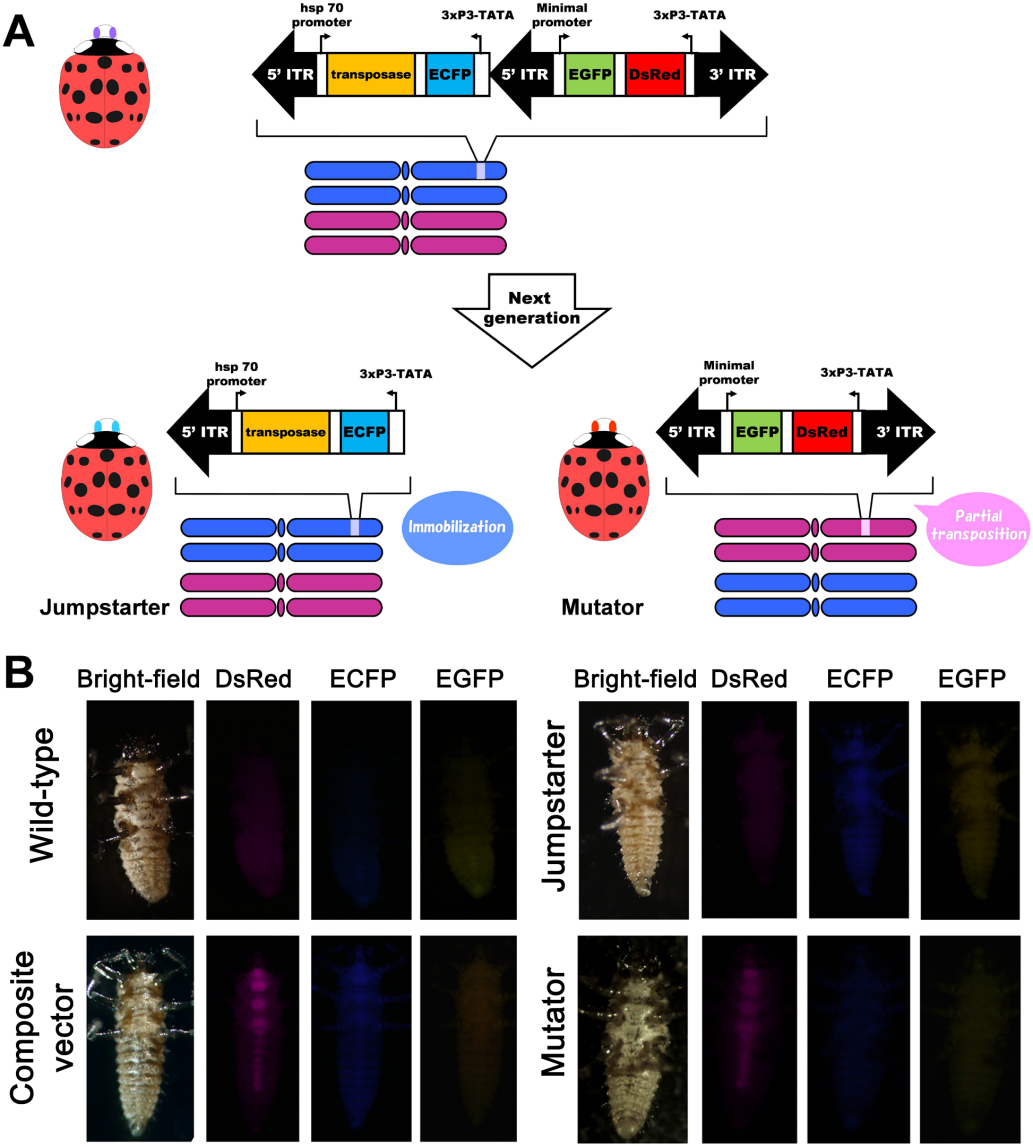
Schematic of composite vector and overview of genomic integration. (A) The left half of the composite vector contains a 5′ ITR, a *transposase* gene under the control of the *Dmhsp70* promoter and the 3xP3-*ECFP* transformation marker, while the right half contains both 5′ and 3′ ITRs, and is marked by both an *EGFP* gene under the control of the *Dmhsp27 *minimal promoter for enhancer trapping and a 3xP3-*DsRed* transformation marker. The *transposase* gene can be immobilized by the precise excision of the sequence between the central 5′ ITR and the 3′ ITR; in this case, larvae expressed ECFP alone. (B) Transformation markers expressed in the CNS of first instar larvae of *H. axyridis* indicate vector integration. From left to right, bright field, DsRed, ECFP and EGFP are presented in wild-type, composite vector (DsRed and ECFP are expressing), Jumpstarter (only ECFP is expressing) and Mutator (only DsRed is expressing). EGFP expression is not detected in all of strains. All panels display a ventral view (anterior uppermost).

The composite vector is designed to produce an immobilized jumpstarter element and a mutator (Fig. 1A). The left half contains a 5′ ITR and a *transposase* gene; the right half contains both 5′ and 3′ ITRs. First, the composite vector is integrated into a chromosome. Transposase acts to remobilize the right half of the vector (5′ and 3′ ITRs) and integrate it elsewhere in the genome. This part is used as a mutator. The remainder, the left half with the 5′ ITR and *transposase* gene, are no longer mobile and remain in the original insertion site. This part is used as a jumpstarter.

In this study, we constructed a *piggyBac*-based transposon vector, pBac(hsp70-transposase)::(3xP3-ECFP)::hsp27-EGFP::(3xP3-DsRed), containing the *piggyBac transposase* gene under the control of *Drosophila heat shock protein 70* (*Dmhsp70*) promoter [26] as the jumpstarter element and the *Enhanced Green Fluorescent Protein* (*EGFP*) gene under the *hsp 27 *minimal promoter as the mutator element (Fig. 1B) to detect a genomic enhancer. The *Enhanced Cyan Fluorescent Protein* (*ECFP*) gene and *Discosoma Red Fluorescent Protein* (*DsRed*) gene under the control of the 3xP3 element, which promotes expression in visual systems and the central nervous system (CNS) [29], were used as transformation markers for the jumpstarter and mutator, respectively. Two types of integration into the genome in G_1_ were obtained by microinjection of the vector into early embryos (G_0_): G_1_ larvae expressing both DsRed and ECFP confirmed integration of the entire composite element using the left end of the 5′ and 3′ ITRs, and larvae expressing DsRed alone indicated integration of the right half of the vector using the middle of the 5′ and 3′ ITRs.

To examine the utility of the vector carrying the composite element, we generated transgenic ladybird beetles. The transformation efficiency of the vector was 2.3% (Table 1) and five G_0_ individuals produced offspring that expressed both DsRed and ECFP (Fig. 1B). Three individuals also gave rise to progeny expressing DsRed alone (Fig. 1B), which were used as a mutator. Single transgenic G_1_ individuals that expressed both ECFP and DsRed markers were crossed with wild-type. In the G_2_ generation, larvae expressing ECFP alone were obtained and used as the potential jumpstarters (Fig. 1B). The precise excision of the right half of the vector was confirmed using inverse polymerase chain reaction (PCR) (data not shown). The jumpstarter element was very stable and has been subsequently maintained for 17 generations over eleven years. Insertions possessing the full composite element could possiblly move as a single unit, or because of the preference for local movement of *piggyBac*, even if the jumpstarter and mutator are no longer a single unit, they could be too close together to detect remobilization via our selection method.

**Table 1:** Transformation efficiency of *piggyBac* vector.

Injected embryos	Hatched larvae	Eclosed adults	Fertile adults	Fertile adults given transgenic insects	Transformation efficiency (%)
914	465	299	219	5	2.3

**Figure 2:**
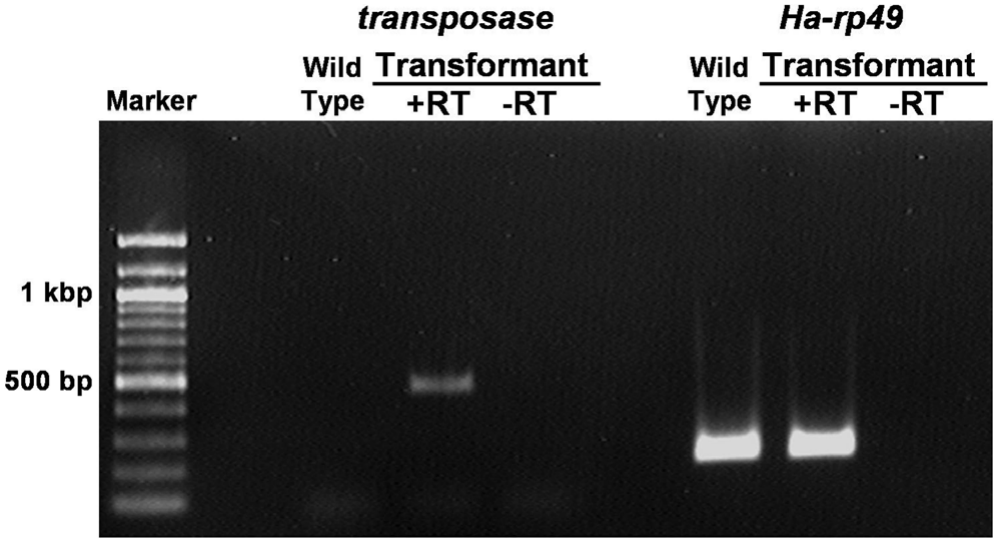
Reverse transcriptase PCR (RT-PCR) analysis of *piggyBac transposase* expression. Total RNA was extracted from transgenic pupae that expressed only the ECFP marker, and from wild-type pupae reared at 25°C. A reaction without reverse transcriptase (-RT) was performed with cDNA synthesis as a negative control. *Harmonia axyridis ribosomal protein 49* (*Ha-rp49*) was used as an internal control.

Next, RT-PCR analysis of the jumpstarter line was performed to confirm expression of *transposase* mRNA. When pupae were reared at a constant temperature of 25°C, *transposase* mRNA was expressed (Fig. 2). This basal expression of the *transposase* gene in the absence of heat shock resulted in sufficient activity for transposition in the germline. To determine the heat shock conditions required for the highest expression of the *transposase* gene by the *Dmhsp70* promoter in *Harmonia*, pupae were exposed to 1-h heat shocks at several temperatures. The highest expression of *transposase* mRNA was observed after a heat shock at 40°C (Figure S1), suggesting that the *Dmhsp70* promoter is heat-inducible in *Harmonia*.

**Figure 3:**
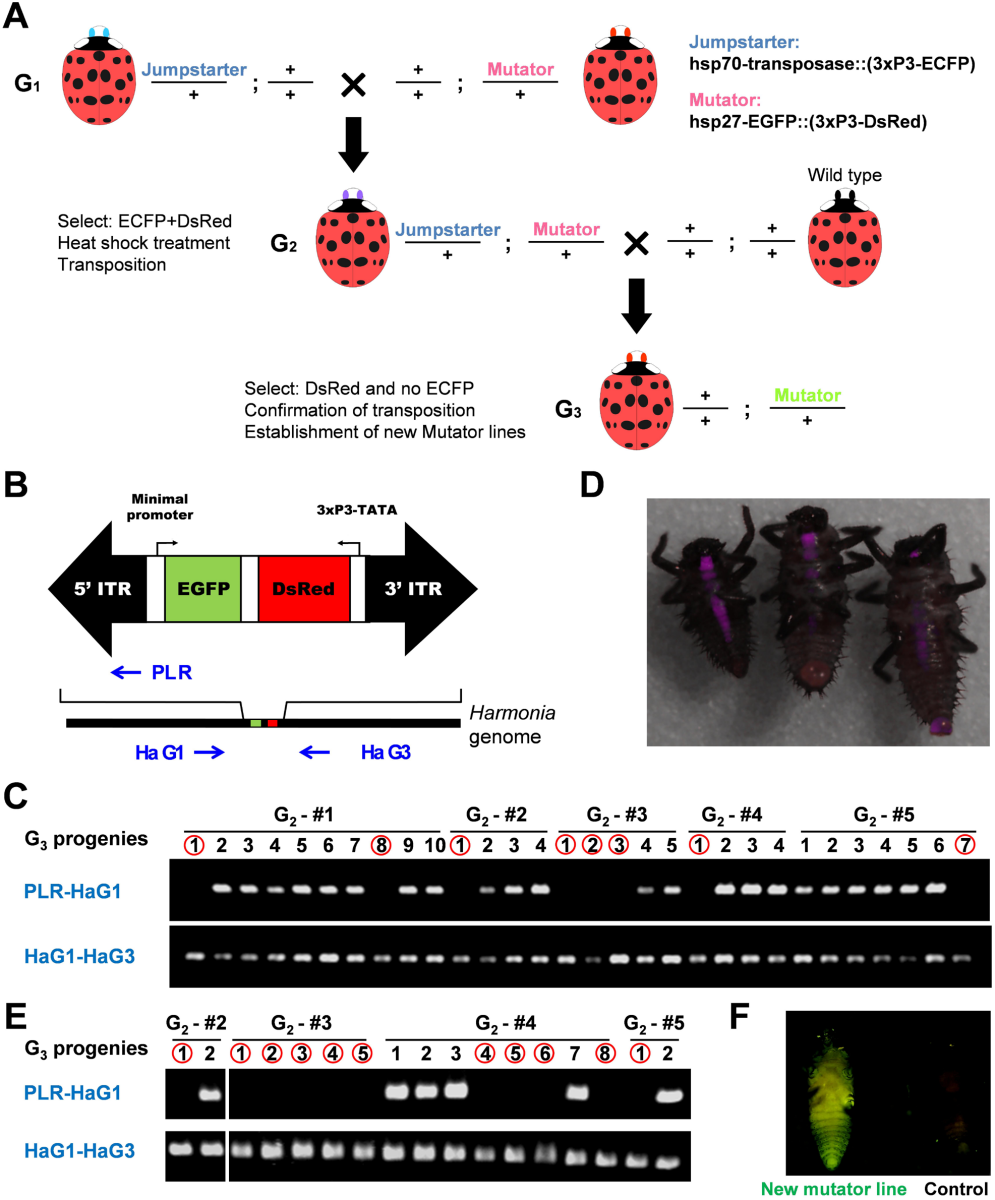
Jumpstarter method in *Harmonia axyridis*. (A) Crossing scheme for jumpstarter method. A mutator line and a jumpstarter line are crossed (G_1_). Newly hatched larvae expressing both ECFP and DsRed transformation markers in the CNS were selected from the progeny (G_2_). Larvae carrying both the mutator and jumpstarter elements were heat shocked to induce *transposase* expression. Newly hatched larvae expressing only the DsRed transformation marker were selected from the progeny (G_3_), and analyzed by PCR for mobilization of the mutator (i.e. integration elsewhere in the genome). Each unique insertion line was used to establish a new mutator line. (B) Schematic representation of PCR method to analyze mobilization of the original mutator element. Primer set HaG1 and PLR was used to detect the original non-mobilized mutator element. If the original mutator element is remobilized, no PCR product will be detected. Primer set HaG1-HaG3 was used as a positive control to detect the homologous, mutator element-free chromosome. (C) Red circles denote progeny with apparent remobilization events. PCR amplification with the PLR-HaG1 primer set was not detected in progeny marked with a red circle, because the original mutator element was mobilized to other genomic sites. These larvae were established as new mutator lines. Each G_2_ female gave at least one newly mobilized progeny. (D) Comparison of DsRed fluorescence between original mutator line (middle) and two new mutator lines (left and right). Compare to original mutator line (middle), two new mutator line larvae show higher (left) or lower (right) expression of DsRed. (E) PCR analysis of mutator element mobilization. Newly hatched G_3_ larvae strongly or weakly expressing DsRed marker compared with original mutator line were selected and subjected to PCR analysis. PCR amplification with the PLR-HaG1 primer set was not detected in progeny marked with a red circle. In this case, detection efficiency of remobilized mutator element is increased compared with random selection (see result in C). (F) An enhancer-trap line of transgenic ladybird beetles. During the generation of mobilized mutator lines using the jumpstarter method, an enhancer expressing GFP throughout the body was detected (left); a wild-type control larva is also shown on the right. Panel displays a ventral view (anterior uppermost).

We successfully implemented the jumpstarter method in the ladybird beetles (Fig. 3A). After crossing the jumpstarter line with the mutator line (Fig. 3A, G_1_ cross), newly hatched larvae carrying both elements were selected according to the presence of transformation markers.

Since all adult males were sterile, mostly owing to the heat sensitivity of spermatogenesis, five females were individually crossed to wild-type males (Fig. 3A, G_2_ cross) and G_3_ progenies selected for expression of DsRed, but absence of CFP expression. Any such progeny should carry only the mutator element and were randomly selected for PCR transposition analysis (Fig. 3B). Jumping rate was 100%, that is, all five G_2_ females gave rise to at least one new insertion line in G_3_ progeny (Fig. 3C). In total, 27% of progeny without a jumpstarter element contained a new insertion of the mutator element; this is on par with the P-element-mediated jumpstarter method used in *Drosophila* [30].

**Figure 4:**
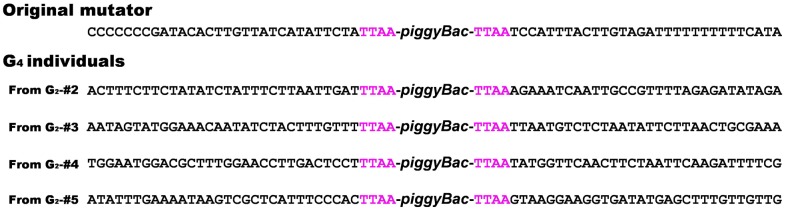
Results of inverse PCR for confirming remobilization of mutator. We performed inverse PCR using extracted DNA from original mutator individual and G_4_ individuals. Each G_4_ individual was derived from G_2_ individuals (#2–5, described in Fig. 3E). Each mutator inserted to different position in each genome.

Further, to increase the efficiency of establishing new mutator lines, we tested the possibility of selecting larvae with new insertions according to the strength of DsRed fluorescence. The position effect acting on genome insertion sites affects expression of the transformation marker, so by selecting larvae with higher or lower DsRed marker expression compared with that of the original mutator (Fig. 3D), we increased the efficiency of selection to 62% (Fig. 3E). In addition to this, we also confirmed remobilization by inverse PCR (Fig. 4). Furthermore, an enhancer-trap line expressing EGFP fluorescence throughout the body was generated (Fig. 3F). Such newly established enhancer-trap line with easily detectable marker also can be used as new mutator line.

However, our examination of several larval stages did not detect EGFP expression in other mutator lines. This suggests that the minimal promoter used in this study was not suitable for enhancer detection in *Harmonia*, as the choice of minimal promoter is crucial in *Bombyx* [31].

As shown in this study, we have successfully established stable transgenic mutator and jumpstarter lines. The standard methods for establishing jumpstarter and mutator lines requires researchers to perform two independent transformations. The strength of the method described here is the ability to generate both jumpstarter and mutator lines with a single transformation. In addition, we demonstrated the potential for the development of various enhancer-trap lines by crossing both transformed lines. This is the first report of a single transformation vector being used to generate separate helper and donor elements for functional analyses in non-model insects. Especially, ladybird beetles are used as study material in both of basic natural science (e.g. color pattern polymorphism) [32], [33] and applied sciences (e.g. biological control) [34]. Also, this method can be applied to the transgenic analyses of any organism using any two-component system.

## Supporting Information

Figure S1 **Effect of differential heat shock temperature on** ***piggyBac transposase*** **expression.** A reaction without reverse transcriptase (-RT) was performed with cDNA synthesis as a negative control. *Harmonia axyridis ribosomal protein 49* (*Ha-rp49*) was used as an internal control.
(DOC)
