# Supplementary material for: Establishment of Transgenic Lines for Jumpstarter Method Using a Composite Transposon Vector in the Ladybird Beetle, Harmonia axyridis
Source: PLoS One. 2014 Jun 24;9(6):e100804. doi: 10.1371/journal.pone.0100804 (PMC4069169; doi:10.1371/journal.pone.0100804)
Supplement: Figure S1 — Effect of differential heat shock temperature on piggyBac transposase expression. A reaction without reverse transcriptase (-RT) was performed with cDNA synthesis as a negative control. Harmonia axyridis ribosomal protein 49 (Ha-rp49) was used as an internal control. (DOC) [file pone.0100804.s001.doc]

**Supplementary Figure**


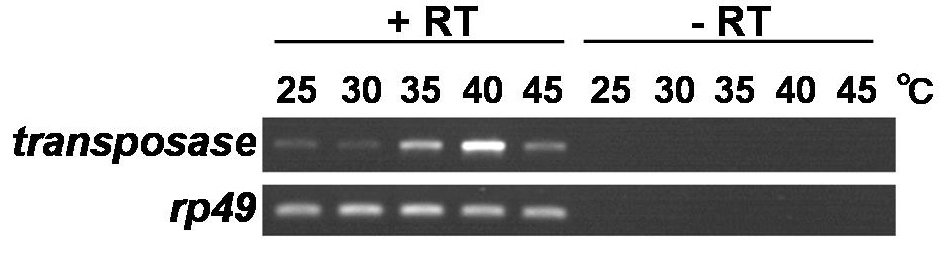


**Figure. S1. Effect of differential heat shock temperature on *piggyBac transposase* expression.**

A reaction without reverse transcriptase (-RT) was performed with cDNA synthesis as a negative control. *Harmonia* *axyridis ribosomal protein 49* (*Ha-rp49*) was used as an internal control.
